# Prognostic significance of preoperative prognostic nutritional index in hepatocellular carcinoma after curative hepatectomy: a meta-analysis and systemic review

**DOI:** 10.3389/fnut.2024.1433528

**Published:** 2024-12-23

**Authors:** Haiyan Zhang, Dan Li, Jing Li

**Affiliations:** Hubei Cancer Hospital, Tongji Medical College, Huazhong University of Science and Technology, Wuhan, China

**Keywords:** prognostic nutritional index, hepatocellular carcinoma, survival, prognosis, meta-analysis

## Abstract

**Background:**

The Prognostic Nutritional Index (PNI), which reflects both nutritional and immune status, has emerged as a potential predictor of survival outcomes in cancer patients. However, its role in forecasting the prognosis of hepatocellular carcinoma (HCC) following curative hepatectomy remains unclear. To further investigate the association between PNI and survival outcomes in HCC patients, we conducted a systematic review and meta-analysis.

**Methods:**

We performed a comprehensive search across Web of Science, PubMed, Embase, Cochrane Library, and China National Knowledge Infrastructure to identify studies evaluating the prognostic value of PNI in HCC following curative hepatectomy. Overall survival (OS), recurrence-free survival (RFS), and disease-free survival (DFS) were extracted as primary outcomes. Pooled hazard ratios (HRs) with 95% confidence intervals (CIs) were calculated using fixed-effect or random-effect models. Additionally, heterogeneity, publication bias, and sensitivity analyses were performed to evaluate the consistency and robustness of the obtained results.

**Results:**

This systematic review and meta-analysis included 19 studies comprising a total of 9,830 patients. The results indicated that higher PNI was significantly associated with longer overall survival (OS) (*n* = 6,812; HR = 1.60; 95% CI: 1.44–1.77; *p* < 0.001) and recurrence-free survival (RFS) (*n* = 8,529; HR = 1.48; 95% CI: 1.30–1.69; *p* < 0.001). There was significant heterogeneity among studies for RFS (*I*^2^ = 56.0%, *p* = 0.004). Subgroup analysis indicated that age, variations in PNI cutoff values and follow-up periods were the primary contributors to this heterogeneity. The trim-and-fill method indicated that publication bias did not impact the OS results, and Egger’s test found no publication bias for RFS (*p* = 0.104). Sensitivity analysis further confirmed the stability of these results.

**Conclusion:**

Preoperative PNI is a significant prognostic indicator in HCC patients undergoing curative hepatectomy, with higher PNI correlating with improved survival outcomes.

**Systematic review registration:**

https://www.crd.york.ac.uk/prospero/display_record.php?ID=CRD42024530150, identifier CRD42024530150.

## 1 Introduction

Hepatocellular carcinoma (HCC) is the most prevalent form of liver cancer, presenting a significant global health burden (1). Despite advancements in diagnostic and therapeutic strategies, the prognosis for HCC patients, especially those undergoing curative hepatectomy, remains variable and frequently uncertain (2). Therefore, identifying reliable prognostic factors for HCC is fundamentally crucial.

The prognosis of HCC is influenced by a number of factors, including tumor diameter, disease stage, liver function, alpha-fetoprotein (AFP), vascular invasion, cirrhosis, hepatitis B or C infection, alcoholic liver disease (1, 3). Notably, markers related to malnutrition and inflammation have proven to be reliable prognostic indicators. High levels of lymphocytes and tumor-infiltrating lymphocytes suggest a potent immune defense against cancer (4). In contrast, elevated neutrophil-to-lymphocyte and platelet-to-lymphocyte ratios, along with programmed cell death-ligand 1 (PD-L1) expression, indicate inflammation and immune escape, which are associated with poorer cancer outcomes (5). Additionally, changes in body mass index, the prognostic nutritional index (PNI), serum albumin (ALB), and C-reactive protein levels crucially reflect a patient’s nutritional and immune status. These indicators significantly impact cancer prognosis by revealing insights into malnutrition, systemic inflammation, and survival expectations (6).

Among various prognostic indicators, PNI has emerged as a potential factor influencing the prognosis of cancer patients, including those with HCC (7). PNI is calculated based on ALB levels and total lymphocyte counts in the blood, with the formula as follow: PNI = 10 × ALB (g/dL) + 0.005 × total lymphocyte count (/mm^3^). Serum ALB, an acute-phase protein, has antioxidant and anti-inflammatory properties which can serve as an important indicator of both nutritional status and systemic inflammation (8). Lymphocytes play a vital role in cell-mediated immunity, inhibiting tumor cell proliferation and invasion through cytokine-mediated cytotoxicity (9, 10). Consequently, a low PNI serves as an indicator of insufficient nutritional and immune function in cancer patients. Various researches have shown that a low preoperative PNI is an independent negative prognostic factor for various digestive system neoplasms, including gastric and colorectal cancers (11), as well as for lung (12), breast (13), ovarian cancers (14), and gastrointestinal stromal tumors (15, 16).

However, the role of PNI in predicting prognosis for patients with HCC remains debated (17–19). Numerous studies suggested that a low PNI served as a prognostic indicator in patients with HCC following surgery (20–22). For example, the study of Hanxin Feng indicated that PNI was a significant prognostic markers for overall survival (OS), but not for disease-free survival (DFS) (23). Conversely, Xiaoxiao Fan’s study revealed that HCC patients with a PNI below 45 had a poor recurrence-free survival (RFS) rate, though this association did not extend to OS (20).

Moreover, multiple meta-analyses have identified the PNI as an independent risk factor for patients with liver cancer post-surgery (20, 24, 25). However, the previous meta-analyses only included studies published up to 2021. Since then, numerous studies on PNI in HCC had been published. Additionally, the value of PNI in peripheral blood may vary with different treatment methods, such as chemotherapy and immunotherapy, an aspect overlooked in previous analyses. In the systematic review, we analyzed the association between preoperative PNI in treatment-naive patients and cancer survival outcomes.

## 2 Materials and methods

### 2.1 Literature search

On April 3, 2024, a comprehensive literature search was conducted across Embase, PubMed, the Cochrane Library, Web of Science, and China National Knowledge Infrastructure (CNKI), without language restrictions. The search strategy incorporated keywords such as “PNI,” “HCC,” “prognosis,” “survival,” and “treatment outcome,” alongside their respective Medical Subject Headings (MeSH) terms. This meta-analysis was designed in accordance with the Preferred Reporting Items for Systematic Reviews and Meta-Analyses (PRISMA) guidelines.

### 2.2 Study selection

Our systematic review aimed to address the following research question: What is the relationship between PNI and cancer survival in patients with HCC? We employed the PICOS (Patient, Intervention, Comparison, Outcome, and Study Design) framework to define selection criteria, as follows: “P” (patient)—patients diagnosed with HCC; “I” (intervention)—not applicable; “C” (comparison)—comparison between groups with high and low PNI; “O” (outcome)—relevant indicators to evaluate the association between preoperative PNI and prognostic outcomes on peripheral blood analysis; and “S” (study design)—prospective and retrospective study. According to the PICOS principles, the inclusion criteria were as follows: (1) Studies evaluated the association between various indicators and predictive outcomes, including PNI; (2) the patients were categorized into high and low PNI groups based on PNI values; (3) studies reported the prognosis of PNI value using multivariate Cox regression analysis. The exclusion criteria were as follows: (1) reviews, case reports, letters, editorials, and meeting abstracts; (2) full text was not available; (3) animal or *in vitro* experiments rather than clinical studies; (4) absence of preoperative PNI measurements prior to curative liver resection; (5) patients who received antitumor therapy before surgery or biopsy, as well as these whose treatment history were unclear; (6) studies that did not directly provide hazard ratios (HR) and 95% confidence intervals (CI).

### 2.3 Data extraction

Three investigators independently extracted data following consistent criteria, with any disagreements resolved through consensus. The data collected included the publication year, first author’s name, country, median age, gender, number of patients, outcome endpoints, PNI cut off value, median follow-up period, and method for estimating HRs with CIs. This meta-analysis focused on three outcome endpoints: OS, RFS, and DFS.

### 2.4 Quality assessment

Three investigators independently assessed the quality of included studies using the Quality in Prognostic Studies (QUIPS) tool. The assessment encompassed: study participation, study attrition, prognostic factor measurement, outcome measurement, study confounding, and statistical analysis. Studies were classified as having a low risk of bias if more than four of these six criteria demonstrated a low risk of bias. Conversely, studies with two or more criteria showing a high risk of bias were categorized as high risk of bias. Studies that did not meet either threshold were classified as having a moderate risk of bias.

### 2.5 Statistical analysis

Statistical analysis was conducted using RevMan 5.4 (Informer Technologies, Los Angeles, CA, USA) and Stata version 14.0 (Stata Corporation, College Station, TX, USA). Pooled HRs and 95% CIs for OS, RFS, and DFS were calculated to evaluate the association between PNI and survival outcomes. Heterogeneity was assessed by the *I*^2^ value derived from the Q test, with *p* < 0.05 or *I*^2^ > 50% indicating significant heterogeneity. Effect models were selected based on the I^2^ and *p* values: a random-effects model was applied if *I*^2^ > 50% or *p* < 0.05; otherwise, a fixed-effect model was applied. Publication bias was evaluated with Egger’s test and the trim-and-fill method. Sensitivity analysis was performed to assess the stability of results by sequentially excluding individual studies.

## 3 Results

### 3.1 Study selection

**Figure 1:**
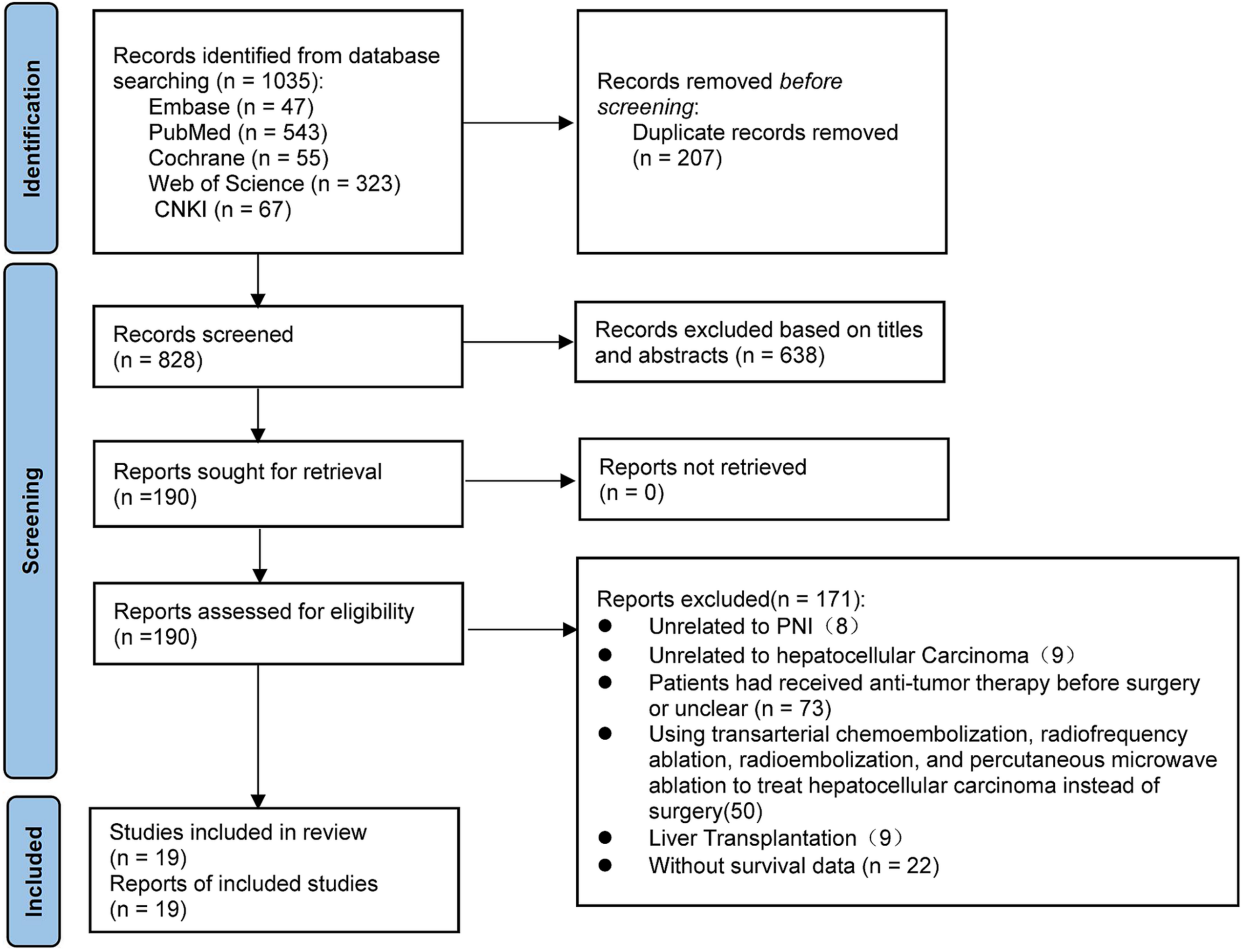
Flow diagram of study selection.

A total of 1,035 papers were initially identified. After removing duplicate literature and reading the title, abstract, and full text according to the study’s inclusion and exclusion criteria, ineligible publications were excluded. Ultimately, 19 studies were identified, comprising 9,830 patients with HCC who underwent curative resection (7, 20, 23, 26–41). These studies were published between 2016 and 2024. The flow diagram for the study selection is presented in Figure 1.

### 3.2 Study characteristics and quality assessment

Table 1 in the meta-analysis presents the characteristics of the included studies, all of which were conducted in Asia, including 14 from China, 1 from Korea, and 4 from Japan, totaling 19 studies. Notably, in one study (40), the correlation between OS and PNI was separately discussed for patients with TNM stage I and TNM stage II. The sample sizes ranged from 100 to 2020 participants, with all studies being retrospective in design. Thirteen studies evaluated the impact of the PNI on OS, 12 on RFS, and 3 on DFS. The studies revealed that the median age of participants ranged from 49.63 to 70.46 years, with a higher prevalence of male participants. The majority of patients were in the early stages of disease, with 7,950 patients classified under the Barcelona Clinic Liver Cancer (BCLC) staging system as stages 0/A and 1,550 in advanced stages B/C/D. Most patients were affected by hepatitis B virus (HBV) or hepatitis C virus (HCV), and all underwent curative surgical excision. Follow-up periods varied, with median durations from 1.9 to 5.3 years. The median cut off values for PNI ranged from 44.35 to 53.95. Preoperative routine blood examinations were conducted, and HRs were calculated using multivariate regression analysis in all studies. According to the QUIPS checklist, 17 studies were assessed as having a low risk of bias, while 2 studies exhibited a moderate risk of bias.

**Table 1:** Characteristics of studies included in the meta-analysis.

No	Years	First author	Country	Sample size	PNI low/high	Age (mean or median, range)	Male/Female	Outcome	Cut off	Follow-up (year)	BCLC		Risk of bias
											0/A	B/C/D	
1	2024	Chengkun Yang	China	1,666	582/1084	<60	1419/247	OS, RFS	46	about 5	1,419	247	L
2	2023	Hikaru Hayashi	Japan	303	150/153	70.47	221/82	OS, RFS	46.2	about 0.2–11.75	221	82	L
3	2022	Wei Qian	China	661	193/468	51	572/89	OS, RFS	45	3 (1.6–3.2)	572	89	L
4	2022	Takashi Matsumoto	Japan	497	116/381	69 (38–87)	374/123	OS	45	4.3	374	123	L
5	2022	Hanxin Feng	China	283	100/183	58 (30–79)	223/60	OS, DFS	48.48	3.3 (0.2–8.9)	223	60	M
6	2021	Meilong Wu	China	88	20/68	NA	62/26	DFS	44.35	about 3	62	26	L
7	2021	Wu meilong	China	73	50/23	NA	57/16	OS	45.65	2.6 (0.2–4.7)	57	16	M
8	2021	Xiaoxiao Fan	China	187	65/122	57 (29–85)	165/22	OS, RFS	45	1.9 (0.1–5)	165	22	L
9	2021	Dong Wang	China	202	NA	50.4 (38.5–62.4)	168/34	OS, RFS	50.25	about 5	168	34	L
10	2021	Yu Saito	Japan	162	86/76	65.1	119/43	RFS	45	2.5 (0.02–8.0)	119	43	L
11	2021	Xie Liang	China	868	230/638	50.5 (38.5–62.6)	727/141	OS, DFS	46	about 3.4–8.3	727	141	L
12	2021	Ho Jeong	Korea	130	77/53	NA	111/19	RFS	52	2.9 (0.2–13.1)	111	19	L
13	2020	Jianxing Zeng	China	2020	1,552/468	51.4 (40.6–62.2)	1,765/255	RFS	53.95	3.9	1,765	255	L
14	2020	Junsheng Yang	China	238	81/157	59.1 (47.8–70.4)	195/43	RFS	48.05	3.1 (0.07–10.1)	195	43	L
15	2020	Z. X. Lin	China	380	189/191	50 (19–80)	333/47	RFS	50	4.1	333	47	L
16	2019	Tingting Zhang	China	401	170/231	52.1 (41.6–62.5)	354/47	OS	48.5	about 10–12.8	354	47	L
17	2019	Paoyuan Huang	China Taiwan	891	441/450	58.5 (46.9–70.1)	694/197	OS, RFS	45	5.3 (2.3–8.3)	694	197	L
18	2017	Yukiyasu Okamura-I	Japan	230	162/68	NA	183/47	OS	52	3.45 (0.5–10)	NA	NA	L
	2017	Yukiyasu Okamura-II	Japan	100	39/61	NA	NA	OS	47	3.45 (0.5–10)	NA	NA	L
19	2016	Sijia Wu	China	450	220/230	49.6 (17–81)	391/59	OS, RFS	48.28	3.8 (0.2–7.7)	391	59	L

OS, overall survival; RFS, recurrence-free survival; DFS, disease-free survival; L, Low-risk; M, Moderate-risk; NA, Not available.

### 3.3 The relation between PNI and OS in HCC patients

**Figure 2:**
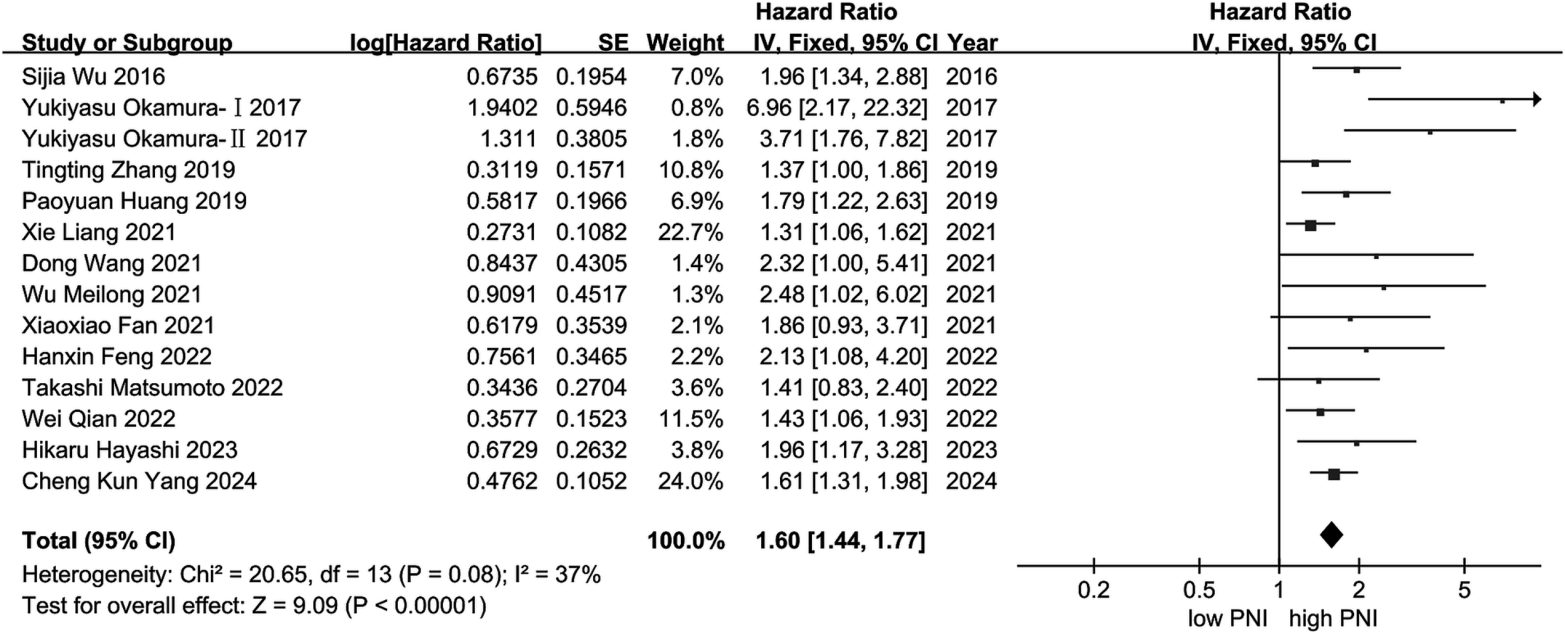
Forest plot of HR with 95% CI for correlation between expression of PNI and OS.

Thirteen studies were included in the meta-analysis of OS. A fixed effect model was used to calculate the pooled HRs and 95% CIs, as the heterogeneity test reported a *p* value of 0.08 and *I*^2^ value of 37.1%. The results showed that patients with higher PNI had significantly longer OS (*n* = 6,812, HR = 1.60; 95% CI: 1.44–1.77; *p* < 0.001) (Figure 2).

### 3.4 The relation between PNI and RFS in HCC patients

**Figure 3:**
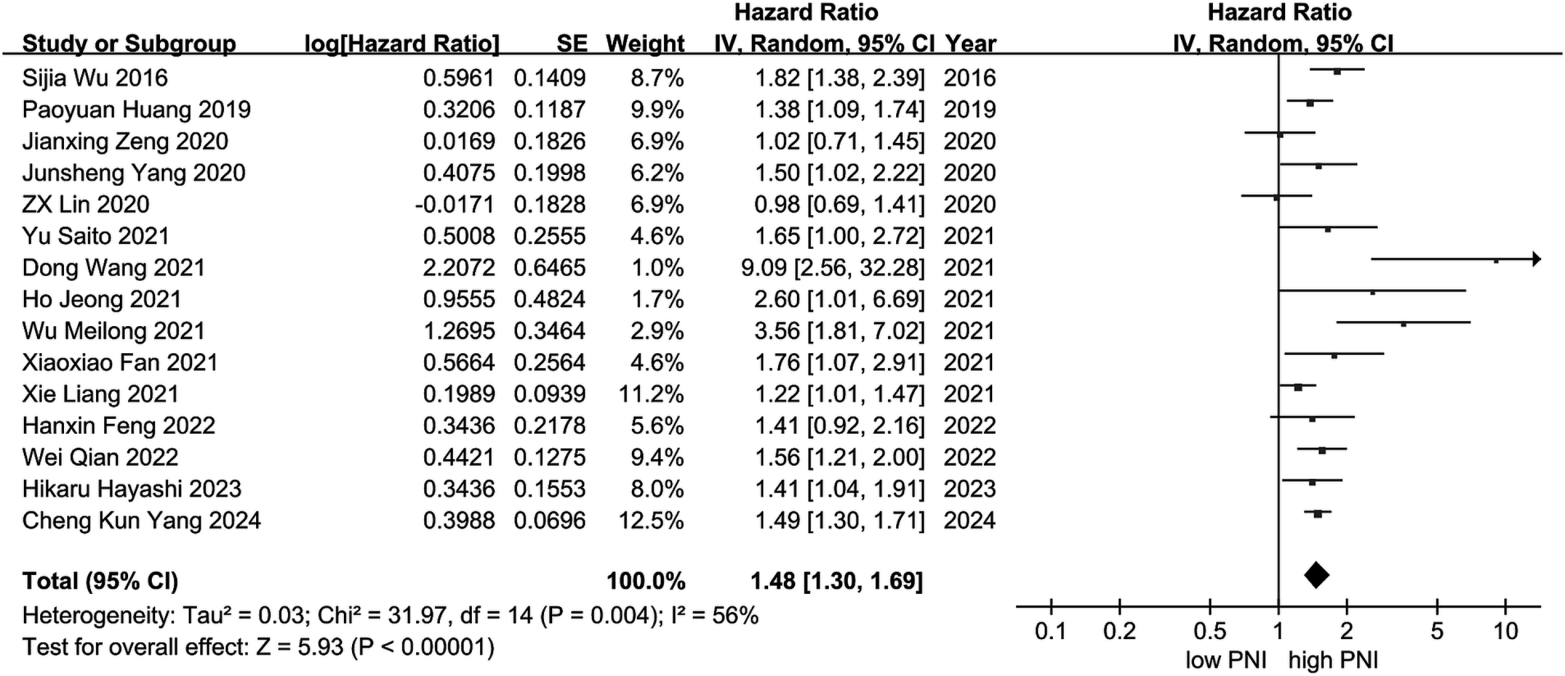
Forest plot of HR with 95% CI for correlation between expression of PNI and RFS.

A total of 12 studies reported the effects of PNI on RFS in the meta-analysis. Besides, 3 articles discussed DFS, which has a similar definition to RFS. Therefore, the HR of DFS was combined with RFS to obtain the final HR for the total 15 studies. A random effects model was used to calculate the pooled HRs and 95% CIs due to the relatively high heterogeneity (*I*^2^ = 56.0%, *p* = 0.004). Our results demonstrated that a higher PNI was associated with improved survival outcomes (*n* = 8,529, HR = 1.48, 95% CI: 1.30–1.69, *p* < 0.001) (Figure 3).

Subgroup analyses of RFS were conducted based on several potential factors (age, sample sizes, cut off value of PNI, follow-up periods, and study quality) to investigate the heterogeneity (Table 2). The result indicated that age, cut off value of PNI and follow-up period were likely contributors to heterogeneity. The heterogeneity for studies with age < 60 (HR =1.41, 95% CI: 1.20–1.66, *p* < 0.001), PNI cut off value > 46 (HR = 1.41, 95% CI: 1.15–1.73, *p* = 0.001), and follow-up period >3 years (HR = 1.31, 95% CI: 1.04–1.64, *p* = 0.019) was notably high, with *I*^2^ values of 59.1, 63.1 and 59.4%, respectively. In contrast, no significant heterogeneity was observed for studies with age ≥ 60 (HR =1.47, 95% CI: 1.13–1.91, *I*^2^ = 0), PNI cut off value ≤46 (HR = 1.56, 95% CI: 1.35–1.80, *I*^2^ = 31.6%), or a follow-up period ≤3 years (HR = 1.64; 95% CI: 1.34–2.00; *I*^2^ = 0).

**Table 2:** Subgroup analysis of RFS included in the meta-analysis.

Subgroup	HR (95% CI)	*p*	Heterogeneity		Studies
			*p*	*I* ^2^	
Age					
≥ 60	1.47 (1.13–1.91)	0.004	0.599	0	2
< 60	1.41 (1.20–1.66)	<0.001	0.009	59.1%	10
NA	2.21 (1.18–4.15)	0.014	0.026	72.6%	3
PNI Low/High					
> 1	1.44 (1.07–1.94)	<0.001	0.123	48.0%	4
< 1	1.45 (1.27–1.65)	0.015	0.034	50.2%	10
NA	9.09 (2.52–32.70)	NA	NA	NA	1
Sample size					
>350	1.36 (1.19–1.56)	<0.001	0.031	56.7%	7
≤350	1.83 (1.40–2.39)	<0.001	0.044	51.5%	8
Cut off of PNI					
>46	1.41 (1.15–1.73)	0.001	0.006	63.1%	9
≤46	1.56 (1.35–1.80)	<0.001	0.199	31.6%	6
Follow-up period					
>3 years	1.31 (1.04–1.64)	0.019	0.043	59.4%	5
≤3 years	1.64 (1.34–2.00)	<0.001	0.761	0	4
NA	1.60 (1.26–2.04)	<0.001	0.003	72.6%	6
Study quality					
Low-risk	1.49 (1.30–1.71)	<0.001	0.003	59.1%	14
Moderate-risk	1.41 (0.92–2.16)	NA	NA	NA	1

HR, hazard ratio; CI, confidence interval; NA, Not available.

### 3.5 Publication bias and sensitivity analysis

**Figure 4:**
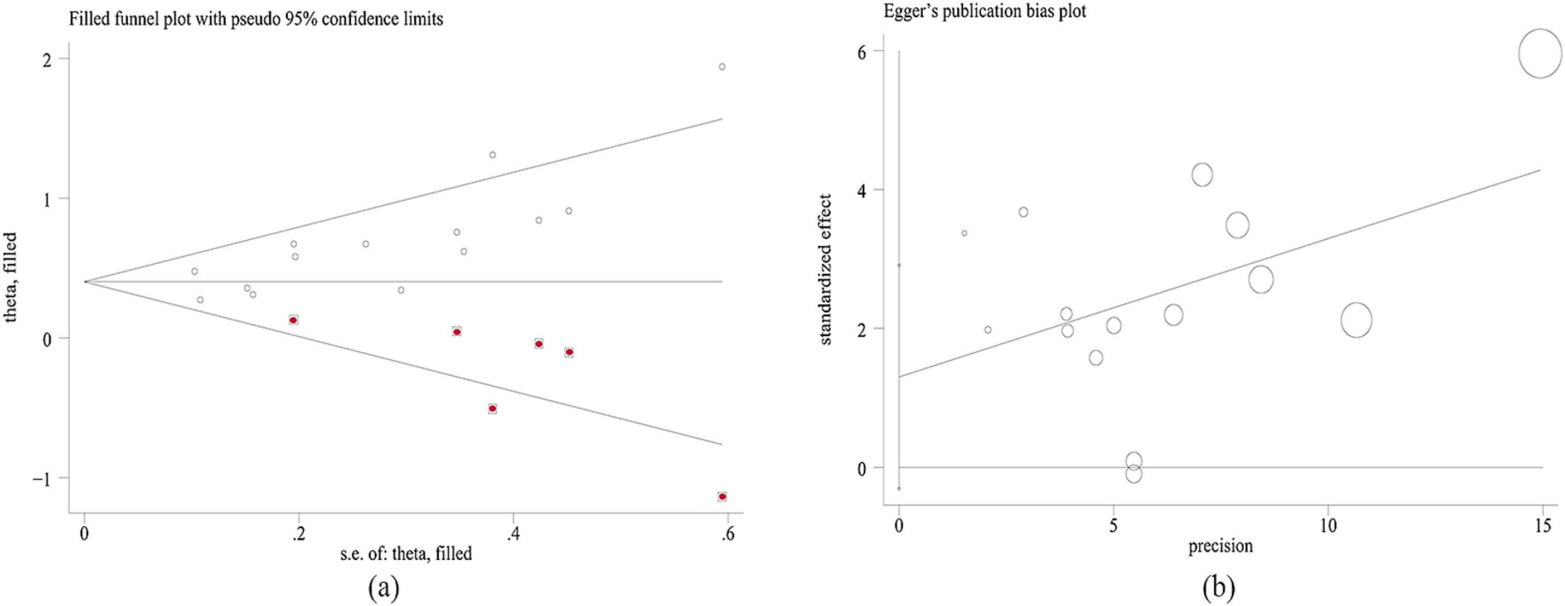
Result of Publication bias. **(a)** Funnel diagram of OS after filled six studies. **(b)** Egger’ s test of RFS.

**Figure 5:**
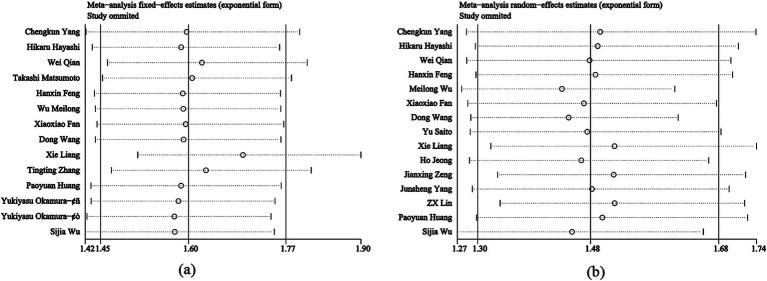
Consequence of sensitivity analysis. **(a)** OS. **(b)** RFS.

Publication bias between studies was conducted using Egger’s test. Results indicated publication bias was found between PNI and OS (*p* = 0.001), while no publication bias was detected between PNI and RFS (*p* = 0.104) (Figure 4b). To further evaluate publication bias for OS, the trim-and-fill method was applied. The addition of six missing studies did not alter the overall effect (HR = 1.495; 95% CI: 1.361–1.642; *p* < 0.001) (Figure 4a), indicating that publication bias for OS did not impact the results and could be ignored. Sensitivity analysis demonstrated that no single study significantly influenced the conclusions of this meta-analysis (Figure 5).

## 4 Discussion

PNI was first proposed by Buzby et al. (42), and later validated by Onodera et al. (43) to predict the surgical risk in gastrointestinal malignancy. Due to its convenience and efficiency, the PNI has been investigated widely, with numerous studies demonstrating that a low PNI is an independent prognostic factor for both short-term postoperative complications and long-term outcomes across various cancers, such as gastric cancer (44), colorectal cancer (44), lung cancer (45), oral cancer (46), biliary tract cancer (47), and so on. In HCC, the PNI was first proposed as a potential prognostic maker by Pinato et al. (48), and its role in HCC treatment and prognosis continues to expand. Pretreatment PNI had been studied across diverse HCC patient groups, including these treated with curative therapies, radiofrequency ablation, microwave ablation (49), sorafenib (50), anti-PD1 therapy (51) and liver transplantation (52). More recently, researches have begun to explore the implications of post-treatment PNI (53). As studies on this topic continue to emerge, there is an urgent need to summarize and analyze the extensive research data to draw meaningful conclusions.

In this review, we comprehensively summarized the literature to date, providing supportive evidence for the prognostic significance of PNI in predicting outcomes for HCC patients following curative hepatectomy. This systematic review and meta-analysis included 19 studies with a total of 9,830 patients. The results indicated that the higher PNI was associated with significantly longer OS (*n* = 6,812, HR = 1.60; 95% CI: 1.45–1.77; *p* < 0.001) and RFS (*n* = 8,529, HR = 1.48, 95% CI: 1.30–1.68, *p* < 0.001), consistent with the previous meta-analyses (20, 24, 25). In the meta-analyses conducted by Guangliu Wu (24) and Xiaoxiao Fan (20), it remained unclear whether the patients received systemic antitumor therapy prior to hepatectomy, and the timing of PNI testing was ambiguous. Notably, Guangliu Wu’s study did not clarify whether PNI was assessed preoperatively or postoperatively (24), and the most recent literature in Xiaoxiao Fan’s study dated back to 2017 (20). Another related meta-analysis suggested that a lower preoperative PNI significantly predicted worse OS and DFS across HCC patients undergoing surgical resection, transcatheter arterial chemoembolization, and non-surgical treatment (25). In our meta-analysis, we only included treatment-naive patients prior to surgery to eliminate the effect of antitumor treatment on PNI. Moreover, the literature included in this paper is relatively new and up to 2024, which provides a more comprehensive understanding of the relationship between PNI and survival outcomes.

The significant statistical heterogeneity was found in RFS. Although the factors of heterogeneity of PNI are very complex, the results of subgroup analyses could partially explain these heterogeneous factors. Subgroup analysis demonstrated that there were significant differences in age, cut-off values of PNI, and follow-up period. Firstly, heterogeneity generation is related to the age. Generally, the elderly are more susceptible to malnutrition due to decreased physiologic function and metabolic level, which in turn may influence PNI. Particularly, the related research also showed that poor nutritional status in HCC patients over 65 years was associated with worse prognoses (54). Consistently, broad age range from 49.6 to 70.5 median years was observed in our study, which may contribute to the heterogeneity. Secondly, the cut off value significantly influences the delineation of specific groupings, which were closely associated with the calculated method. Indeed, three different sources of cut-off value were involved among all the included articles with “previous literature” (7, 20, 29), “survminer” package (30, 35), and ROC data. Thirdly, the follow-up period is also a source of heterogeneity. One study focused on the psoas muscle index (PMI), an indicator similar to PNI, indicated that PMI was an independent prognostic factor for 1-year treatment outcomes but not effective for predicting 6-month outcomes (55). Unfortunately, the absence of precise median follow-up times in six of the studies included in this paper limited the potential for further in-depth analysis. Additionally, differences in multivariate analysis models may also contribute to heterogeneity, as models based on different postoperative inflammatory indicators and clinicopathological factors exhibit varying HRs and 95% CIs (30).

The PNI incorporates measurements, such as ALB levels and lymphocyte count, that reflect both nutritional and immunological status. ALB (56) helps regulate blood volume and pressure, crucial for transporting nutrients, hormones, and immune cells (56). It’s also linked to cancer prognosis, particularly in patients with HCC (57, 58). Lymphocytes, another part of the PNI, can prevent tumor growth and recurrence by supporting immune function (59). Additionally, a low PNI is associated with poor survival rates in metastatic intrahepatic cholangiocarcinoma (ICC) (6). Moreover, it also helps predict outcomes for patients undergoing immunotherapy (60), targeted therapy (50), and radiochemotherapy (61, 62).

There are several reasons why a low preoperative PNI may be associated with a poor prognosis in patients with HCC following curative hepatectomy. Firstly, the PNI serves as an indicator of nutritional status. A low preoperative PNI suggests a compromised nutritional state, which negatively impacts prognosis. Secondly, the study of Pinato noted that PNI correlated significantly with raised AFP, liver functional reserve, and the presence of portal vein thrombosis, suggesting that a high-risk PNI correlated with a more aggressive disease phenotype (48). Additionally, both ALB levels and lymphocyte counts could explain the phenomenon. ALB levels are linked to liver function and have been correlated with survival outcomes across various cancer types, including HCC. The lymphocyte count, an accessible and cost-effective biomarker of inflammation, plays a crucial role in assessing immune function and infection status.

Besides the PNI, patient prognosis may also be influenced by other factors, such as TNM staging (63), BCLC staging (26), age, sex (64), follow-up time (55). The study found that while PNI did not predict OS in HCC cases generally (HR = 1.855, 95% CI: 0.927–3.711; *p* = 0.081), it was an independent prognostic factor for OS in HCC patients who underwent curative hepatectomy at TNM stage I (HR = 2.305, 95% CI: 1.008–5.268; *p* = 0.048) (20). Unfortunately, most of the articles included in this study did not provide staging information, leaving insufficient data for further analysis. Across all 19 studies included, the number of BCLC (0/A) stage patients was greater than that of BCLC (B/C/D) stage patients, and the number of male patients was greater than that of female patients, which makes the conclusions of this paper more applicable to early-stage male liver cancer patients. Notably, most of the patients included had HBV, and antiviral treatment had a considerable impact on the conclusions, as it is known to produce biochemical and virological improvements in chronic HBV patients, including elevated serum ALB levels and increased peripheral T-lymphocyte counts (65). Studies shown that the use of antiviral treatment was associated with higher PNl (66). However, the included studies did not specify whether patients received HBV treatment or provide details of the treatment regimen, limiting the investigation of the relationship between antiviral treatment and PNI.

There were limitations in this meta-analysis. Firstly, we were unable to perform a subgroup analysis for each TNM stage and gender because of the limited number of included studies. Secondly, the cut off value of PNI was not completely consistent between studies, leading to the potential sources of heterogeneity. Finally, all the studies included were retrospective studies, lacking the prospective study. Additionally, all the studies are based in Asia. The lack of research from Europe and America means that the conclusions are only applicable to Asian patients.

## 5 Conclusion

In summary, this meta-analysis and systematic review endeavor to provide a definitive assessment of the prognostic significance of the preoperative PNI in HCC patients undergoing curative hepatectomy. Moreover, based on the conclusion, we speculated that HCC patients could benefit from preoperative treatment, such as enteral nutrition support and preoperative non-steroid anti-inflammatory drugs, to help HCC patients reach a satisfied PNI value.

## Data Availability

The original contributions presented in the study are included in the article/supplementary material, further inquiries can be directed to the corresponding author.

## References

[1] Wang Y Deng B. Hepatocellular carcinoma: molecular mechanism, targeted therapy, and biomarkers. Cancer Metastasis Rev. (2023) 42:629–52. doi: 10.1007/s10555-023-10084-4, PMID: 36729264

[2] Ganesan P Kulik LM. Hepatocellular carcinoma. Clin Liver Dis. (2023) 27:85–102. doi: 10.1016/j.cld.2022.08.004, PMID: 36400469

[3] Piñero F Dirchwolf M Pessôa MG. Biomarkers in hepatocellular carcinoma: diagnosis, prognosis and treatment response assessment. Cells. (2020) 9:1370. doi: 10.3390/cells9061370, PMID: 32492896 PMC7349517

[4] Nie H He T Wang L Zhang L. Expression and prognostic value of tumor-infiltrating lymphocytes and PD-L1 in hepatocellular carcinoma. Onco Targets Ther. (2021) 14:1377–85. doi: 10.2147/ott.S289720, PMID: 33658801 PMC7920601

[5] Zheng J Cai J Li H Zeng K He L Fu H . Neutrophil to lymphocyte ratio and platelet to lymphocyte ratio as prognostic predictors for hepatocellular carcinoma patients with various treatments: a Meta-analysis and systematic review. Cell Physiol Biochem. (2017) 44:967–81. doi: 10.1159/000485396, PMID: 29179180

[6] Zhang C Wang H Ning Z Xu L Zhuang L Wang P . Prognostic nutritional index serves as a predictive marker of survival and associates with systemic inflammatory response in metastatic intrahepatic cholangiocarcinoma. Onco Targets Ther. (2016) 9:6417–23. doi: 10.2147/ott.S112501, PMID: 27799789 PMC5077274

[7] Yang C-K Huang K-T Qin W Wu Q-Y Huang X-L Peng K . Prognostic value of geriatric nutritional risk index and prognostic nutritional index in hepatocellular carcinoma. Clin Nutr ESPEN. (2024) 59:355–64. doi: 10.1016/j.clnesp.2023.12.148, PMID: 38220397

[8] Erstad BL. Serum albumin levels: who needs them? Ann Pharmacother. (2021) 55:798–804. doi: 10.1177/1060028020959348, PMID: 32909438

[9] Sohda M Sakai M Yamaguchi A Watanabe T Nakazawa N Ubukata Y . Pre-treatment CRP and albumin determines prognosis for Unresectable advanced Oesophageal Cancer. In Vivo. (2022) 36:1930–6. doi: 10.21873/invivo.12914, PMID: 35738616 PMC9301391

[10] Buck M Zhang L Halasz NA Hunter T Chojkier M. Nuclear export of phosphorylated C/EBPbeta mediates the inhibition of albumin expression by TNF-alpha. EMBO J. (2001) 20:6712–23. doi: 10.1093/emboj/20.23.6712, PMID: 11726507 PMC125761

[11] Zhang LL Ma WB Qiu ZD Kuang TR Wang KP Hu BH . Prognostic nutritional index as a prognostic biomarker for gastrointestinal cancer patients treated with immune checkpoint inhibitors. Front Immunol. (2023) 14:12. doi: 10.3389/fimmu.2023.1219929, PMID: 37545502 PMC10401046

[12] Peng L Wang Y Liu F Qiu X Zhang X Fang C . Peripheral blood markers predictive of outcome and immune-related adverse events in advanced non-small cell lung cancer treated with PD-1 inhibitors. Cancer Immunol Immunother. (2020) 69:1813–22. doi: 10.1007/s00262-020-02585-w, PMID: 32350592 PMC7413896

[13] Peng P Chen L Shen Q Xu Z Ding X. Prognostic nutritional index (PNI) and controlling nutritional status (CONUT) score for predicting outcomes of breast cancer: a systematic review and meta-analysis. Pak J Med Sci. (2023) 39:1535–41. doi: 10.12669/pjms.39.5.7781, PMID: 37680798 PMC10480717

[14] Dai Y Liu M Lei L Lu S. Prognostic significance of preoperative prognostic nutritional index in ovarian cancer: a systematic review and meta-analysis. Medicine. (2020) 99:e21840. doi: 10.1097/md.0000000000021840, PMID: 32957308 PMC7505367

[15] Kang N Gu H Ni Y Wei X Zheng S. Prognostic and clinicopathological significance of the prognostic nutritional index in patients with gastrointestinal stromal tumours undergoing surgery: a meta-analysis. BMJ Open. (2022) 12:e064577. doi: 10.1136/bmjopen-2022-064577, PMID: 36456008 PMC9717127

[16] Taniai T Furukawa K Igarashi Y Shirai Y Haruki K Onda S . Dynamics of the prognostic nutritional index in preoperative chemotherapy in patients with colorectal liver metastases. Surg Oncol. (2023) 49:101966. doi: 10.1016/j.suronc.2023.101966, PMID: 37419043

[17] Li Q Chen C Zhang J Wu H Qiu YH Song TQ . Prediction efficacy of prognostic nutritional index and albumin-bilirubin grade in patients with intrahepatic cholangiocarcinoma after radical resection: a multi-institutional analysis of 535 patients. Front Oncol. (2021) 11:11. doi: 10.3389/fonc.2021.769696, PMID: 34956888 PMC8702533

[18] Okamura Y Ashida R Ito T Sugiura T Mori K Uesaka K. Preoperative neutrophil to lymphocyte ratio and prognostic nutritional index predict overall survival after hepatectomy for hepatocellular carcinoma. World J Surg. (2015) 39:1501–9. doi: 10.1007/s00268-015-2982-z, PMID: 25670038

[19] Zhou J Yang D. Diagnostic value of OPNI in hepatocellular carcinoma. Oncology. (2023) 101:481–90. doi: 10.1159/000530319, PMID: 37454651

[20] Fan XX Chen GQ Li YR Shi ZQ He LF Zhou DZ . The preoperative prognostic nutritional index in hepatocellular carcinoma after curative hepatectomy: a retrospective cohort study and Meta-analysis. J Investig Surg. (2021) 34:826–33. doi: 10.1080/08941939.2019.1698679, PMID: 31818159

[21] Wang Y Li X Yu J Cheng Z Hou Q Liang P. Prognostic nutritional index in hepatocellular carcinoma patients with hepatitis B following US-guided percutaneous microwave ablation: a retrospective study with 1, 047 patients. Front Surg. (2022) 9:878737. doi: 10.3389/fsurg.2022.878737, PMID: 35846958 PMC9276976

[22] Tanemura A Mizuno S Hayasaki A Gyoten K Fujii T Iizawa Y . Onodera's prognostic nutritional index is a strong prognostic indicator for patients with hepatocellular carcinoma after initial hepatectomy, especially patients with preserved liver function. BMC Surg. (2020) 20:261. doi: 10.1186/s12893-020-00917-2, PMID: 33129309 PMC7603728

[23] Feng HX Xu F Zhao Y Jin TQ Liu JB Li R . Prognostic value of combined inflammatory and nutritional biomarkers in HCC within the Milan criteria after hepatectomy. Front Oncol. (2022) 12:9. doi: 10.3389/fonc.2022.947302, PMID: 36132141 PMC9483162

[24] Wu GL Zhou XY Yang XM Yin RJ. Prognostic evaluation of prognostic nutritional index for patients with hepatectomy for primary hepatocellular carcinoma: a Meta-analysis. Chin Evid Nurs. (2024) 10:392–5. doi: 10.12102/j.issn.2095-8668.2024.03.003

[25] Man Z Pang Q Zhou L Wang Y Hu X Yang S . Prognostic significance of preoperative prognostic nutritional index in hepatocellular carcinoma: a meta-analysis. HPB. (2018) 20:888–95. doi: 10.1016/j.hpb.2018.03.019, PMID: 29853431

[26] Hayashi H Shimizu A Kubota K Notake T Masuo H Yoshizawa T . Combination of sarcopenia and prognostic nutritional index to predict long-term outcomes in patients undergoing initial hepatectomy for hepatocellular carcinoma. Asian J Surg. (2023) 46:816–23. doi: 10.1016/j.asjsur.2022.07.122, PMID: 35961897

[27] Qian W Xiao-Jian J Jun H Liang L Xiao-Yong C. Comparison of the value of multiple preoperative objective nutritional indices for the evaluation of prognosis after hepatectomy for hepatocellular carcinoma. Nutr Cancer. (2022) 74:3217–27. doi: 10.1080/01635581.2022.2069276, PMID: 35533004

[28] Qu Z Lu YJ Feng JW Chen YX Shi LQ Chen J . Preoperative prognostic nutritional index and neutrophil-to-lymphocyte ratio predict survival outcomes of patients with hepatocellular carcinoma after curative resection. Front Oncol. (2022) 11:10. doi: 10.3389/fonc.2021.823054, PMID: 35155212 PMC8831760

[29] Matsumoto T Kitano Y Imai K Kinoshita S Sato H Shiraishi Y . Clinical significance of preoperative inflammation-based score for the prognosis of patients with hepatocellular carcinoma who underwent hepatectomy. Surg Today. (2022) 52:1008–15. doi: 10.1007/s00595-021-02427-x, PMID: 35083547

[30] Wu ML Yang SZ Feng XB Li CQ Liu XC Zhang ZY . Combining preoperative and postoperative inflammatory indicators can better predict the recurrence of hepatocellular carcinoma after partial hepatectomy. J Inflamm Res. (2021) 14:3231–45. doi: 10.2147/jir.S316177, PMID: 34285546 PMC8286132

[31] Wang D Hu X Xiao L Long G Yao L Wang ZM . Prognostic nutritional index and systemic immune-inflammation index predict the prognosis of patients with HCC. J Gastrointest Surg. (2021) 25:421–7. doi: 10.1007/s11605-019-04492-7, PMID: 32026332 PMC7904713

[32] Saito Y Imura S Morine Y Ikemoto T Yamada S Shimada M. Preoperative prognostic nutritional index predicts short- and long-term outcomes after liver resection in patients with hepatocellular carcinoma. Oncol Lett. (2021) 21:153. doi: 10.3892/ol.2020.12414, PMID: 33552271 PMC7798109

[33] Liang X Liangliang X Peng W Tao Y Jinfu Z Ming Z . Combined prognostic nutritional index and albumin-bilirubin grade to predict the postoperative prognosis of HBV-associated hepatocellular carcinoma patients. Sci Rep. (2021) 11:14624. doi: 10.1038/s41598-021-94035-5, PMID: 34272447 PMC8285529

[34] Jeong H Kim KH Jo S Song S. Impact of prognostic nutritional index on the recurrence of hepatocellular carcinoma after a curative resection. Ann Hepatobiliary Pancreat Surg. (2021) 25:456–61. doi: 10.14701/ahbps.2021.25.4.456, PMID: 34845116 PMC8639306

[35] Zeng JX Zeng JH Wu QL Lin KY Zeng JY Guo PF . Novel inflammation-based prognostic nomograms for individualized prediction in hepatocellular carcinoma after radical resection. Ann Transl Med. (2020) 8:1061. doi: 10.21037/atm-20-1919, PMID: 33145280 PMC7575986

[36] Yang J Bao Y Chen W Duan Y Sun D. Nomogram based on systemic immune inflammation index and prognostic nutrition index predicts recurrence of hepatocellular carcinoma after surgery. Front Oncol. (2020) 10:551668. doi: 10.3389/fonc.2020.551668, PMID: 33163397 PMC7591400

[37] Lin ZX Ruan DY Jia CC Wang TT Cheng JT Huang HQ . Controlling nutritional status (CONUT) score-based nomogram to predict overall survival of patients with HBV-associated hepatocellular carcinoma after curative hepatectomy. Clin Transl Oncol. (2020) 22:370–80. doi: 10.1007/s12094-019-02137-4, PMID: 31201606

[38] Zhang T Liu Z Zhao X Mao Z Bai L. A novel prognostic score model based on combining systemic and hepatic inflammation markers in the prognosis of HBV-associated hepatocellular carcinoma patients. Artif Cells Nanomed Biotechnol. (2019) 47:2246–55. doi: 10.1080/21691401.2019.1573174, PMID: 31169437

[39] Huang PY Wang CC Lin CC Lu SN Wang JH Hung CH . Predictive effects of inflammatory scores in patients with BCLC 0-a hepatocellular carcinoma after hepatectomy. J Clin Med. (2019) 8:1676. doi: 10.3390/jcm8101676, PMID: 31614976 PMC6832545

[40] Okamura Y Sugiura T Ito T Yamamoto Y Ashida R Uesaka K. The optimal cut-off value of the preoperative prognostic nutritional index for the survival differs according to the TNM stage in hepatocellular carcinoma. Surg Today. (2017) 47:986–93. doi: 10.1007/s00595-017-1491-0, PMID: 28315008

[41] Wu SJ Lin YX Ye H Li FY Xiong XZ Cheng NS. Lymphocyte to monocyte ratio and prognostic nutritional index predict survival outcomes of hepatitis B virus-associated hepatocellular carcinoma patients after curative hepatectomy. J Surg Oncol. (2016) 114:202–10. doi: 10.1002/jso.24297, PMID: 27199001

[42] Buzby GP Mullen JL Matthews DC Hobbs CL Rosato EF. Prognostic nutritional index in gastrointestinal surgery. Am J Surg. (1980) 139:160–7. doi: 10.1016/0002-9610(80)90246-9, PMID: 7350839

[43] Onodera T Goseki N Kosaki G. Prognostic nutritional index in gastrointestinal surgery of malnourished cancer patients. Nihon Geka Gakkai Zasshi. (1984) 85:1001–5. 6438478

[44] Liu XR Wang LL Zhang B Liu XY Li ZW Kang B . The advanced lung cancer inflammation index is a prognostic factor for gastrointestinal cancer patients undergoing surgery: a systematic review and meta-analysis. World J Surg Oncol. (2023) 21:81. doi: 10.1186/s12957-023-02972-4, PMID: 36879283 PMC9987069

[45] Hayasaka K Shiono S Suzuki K Endoh M Okada Y. Postoperative prognostic nutritional index as a prognostic factor after non-small cell lung cancer surgery. Gen Thorac Cardiovasc Surg. (2020) 68:1163–71. doi: 10.1007/s11748-020-01366-7, PMID: 32328993

[46] Dai ML Sun QJ. Prognostic and clinicopathological significance of prognostic nutritional index (PNI) in patients with oral cancer: a meta-analysis. Aging. (2023) 15:33–3. doi: 10.18632/aging.204576: PMC10042682 36897190

[47] Lv XY Zhang ZX Yuan WB. Pretreatment prognostic nutritional index (PNI) as a prognostic factor in patients with biliary tract Cancer: a meta-analysis. Nutr Cancer. (2021) 73:1872–81. doi: 10.1080/01635581.2020.1817955, PMID: 32933337

[48] Pinato DJ North BV Sharma R. A novel, externally validated inflammation-based prognostic algorithm in hepatocellular carcinoma: the prognostic nutritional index (PNI). Br J Cancer. (2012) 106:1439–45. doi: 10.1038/bjc.2012.92, PMID: 22433965 PMC3326674

[49] Ryu T Takami Y Wada Y Sasaki S Saitsu H. Predictive impact of the prognostic nutritional index in early-staged hepatocellular carcinoma after operative microwave ablation. Asian J Surg. (2022) 45:202–7. doi: 10.1016/j.asjsur.2021.04.043, PMID: 34078578

[50] Gulmez A Harputluoglu H. Advanced hepatocellular Cancer treated with Sorafenib and novel inflammatory markers. J Gastrointest Cancer. (2023) 54:11–9. doi: 10.1007/s12029-021-00789-6, PMID: 35119620

[51] Kang X Wang J Kang X Bai L. Predictive value of prognostic nutritional index (PNI) in recurrent or unresectable hepatocellular carcinoma received anti-PD1 therapy. BMC Cancer. (2023) 23:787. doi: 10.1186/s12885-023-11166-w, PMID: 37612634 PMC10463676

[52] Kornberg A Kaschny L Kornberg J Friess H. Preoperative prognostic nutritional index may be a strong predictor of hepatocellular carcinoma recurrence following liver transplantation. J Hepatocell Carcinoma. (2022) 9:649–60. doi: 10.2147/jhc.S366107, PMID: 35923612 PMC9342250

[53] Pravisani R Mocchegiani F Isola M Lorenzin D Adani GL Cherchi V . Postoperative trends and prognostic values of inflammatory and nutritional biomarkers after liver transplantation for hepatocellular carcinoma. Cancers. (2021) 13:513. doi: 10.3390/cancers13030513, PMID: 33572776 PMC7866292

[54] Li L Wang H Yang J Jiang L Yang J Wu H . Geriatric nutritional risk index predicts prognosis after hepatectomy in elderly patients with hepatitis B virus-related hepatocellular carcinoma. Sci Rep. (2018) 8:12561. doi: 10.1038/s41598-018-30906-8, PMID: 30135506 PMC6105611

[55] Luo N Li H Luo Y Hu P Liang L Zhang R . Prognostic significance of psoas muscle index in male hepatocellular carcinoma patients treated with immune checkpoint inhibitors and tyrosine kinase inhibitors. Hum Vaccin Immunother. (2023) 19:2258567. doi: 10.1080/21645515.2023.2258567, PMID: 37728115 PMC10512869

[56] Zheng M. Serum albumin: a pharmacokinetic marker for optimizing treatment outcome of immune checkpoint blockade. J Immunother Cancer. (2022) 10:e005670. doi: 10.1136/jitc-2022-005670, PMID: 36600664 PMC9772729

[57] Jeng LB Chan WL Teng CF. Prognostic significance of serum albumin level and albumin-based mono- and combination biomarkers in patients with hepatocellular carcinoma. Cancers. (2023) 15:1005. doi: 10.3390/cancers15041005, PMID: 36831351 PMC9953807

[58] Wang L Li Q Zhang J Lu J. A novel prognostic scoring model based on albumin and γ-Glutamyltransferase for hepatocellular carcinoma prognosis. Cancer Manag Res. (2019) 11:10685–94. doi: 10.2147/cmar.S232073, PMID: 31920379 PMC6934113

[59] Wang J Zhou D Dai Z Li X. Association between systemic immune-inflammation index and diabetic depression. Clin Interv Aging. (2021) 16:97–105. doi: 10.2147/cia.S285000, PMID: 33469277 PMC7810592

[60] Tada T Kumada T Hiraoka A Kariyama K Tani J Hirooka M . Nutritional status is associated with prognosis in patients with advanced Unresectable hepatocellular carcinoma treated with Atezolizumab plus bevacizumab. Oncology. (2023) 101:270–82. doi: 10.1159/000527676, PMID: 36455517

[61] Rimini M Kang W Burgio V Persano M Aoki T Shimose S . Validation of the easy-to-use lenvatinib prognostic index to predict prognosis in advanced hepatocellular carcinoma patients treated with lenvatinib. Hepatol Res. (2022) 52:1050–9. doi: 10.1111/hepr.13824, PMID: 35960789

[62] Tohme S Chidi AP Sud V Tsung A. Prognostic nutritional index is associated with survival in patients with Unresectable hepatocellular carcinoma treated with Radioembolization. J Vasc Interv Radiol. (2017) 28:470–2. doi: 10.1016/j.jvir.2016.10.016, PMID: 28231927

[63] Liu CX Zhao HR Zhang RJ Guo ZM Wang P Qu ZW. Prognostic value of nutritional and inflammatory markers in patients with hepatocellular carcinoma who receive immune checkpoint inhibitors. Oncol Lett. (2023) 26:437. doi: 10.3892/ol.2023.14024, PMID: 37664652 PMC10472048

[64] Rich NE Murphy CC Yopp AC Tiro J Marrero JA Singal AG. Sex disparities in presentation and prognosis of 1110 patients with hepatocellular carcinoma. Aliment Pharmacol Ther. (2020) 52:701–9. doi: 10.1111/apt.15917, PMID: 32598091 PMC7655123

[65] Kim JH Park JW Koh DW Lee WJ Kim CM. Efficacy of lamivudine on hepatitis B viral status and liver function in patients with hepatitis B virus-related hepatocellular carcinoma. Liver Int. (2009) 29:203–7. doi: 10.1111/j.1478-3231.2008.01828.x, PMID: 18662281

[66] Chan AWH Chan SL Wong GLH Wong VWS Chong CCN Lai PBS . Prognostic nutritional index (PNI) predicts tumor recurrence of very early/early stage hepatocellular carcinoma after surgical resection. Ann Surg Oncol. (2015) 22:4138–48. doi: 10.1245/s10434-015-4516-1, PMID: 25801356

